# InspectorORF: a tool for visualizing Ribo-Seq and additional genomic or transcriptomic data

**DOI:** 10.1093/bioadv/vbag031

**Published:** 2026-01-27

**Authors:** Eilidh L Ward, Isabel Birds, Mary J O’Connell, David R Westhead, Julie L Aspden

**Affiliations:** School of Molecular and Cellular Biology, Faculty of Biological Sciences, University of Leeds, Leeds LS2 9JT, United Kingdom; LeedsOmics, University of Leeds, Leeds, LS2 9DA, United Kingdom; School of Molecular and Cellular Biology, Faculty of Biological Sciences, University of Leeds, Leeds LS2 9JT, United Kingdom; LeedsOmics, University of Leeds, Leeds, LS2 9DA, United Kingdom; School of Biological Sciences, Faculty of Biology Medicine and Health, University of Manchester, Manchester, M13 9PL, United Kingdom; Natural History Museum London, London, SW7 5BD, United Kingdom; School of Molecular and Cellular Biology, Faculty of Biological Sciences, University of Leeds, Leeds LS2 9JT, United Kingdom; Leeds Institute of Data Analytics (LIDA), University of Leeds, Leeds LS2 9DA, United Kingdom; School of Molecular and Cellular Biology, Faculty of Biological Sciences, University of Leeds, Leeds LS2 9JT, United Kingdom; LeedsOmics, University of Leeds, Leeds, LS2 9DA, United Kingdom; Astbury Centre for Structural Molecular Biology, University of Leeds, Leeds LS2 9JT, United Kingdom

## Abstract

**Motivation:**

The advent of ribosome profiling (an adaptation of RNA sequencing) to determine the translatome, has led to a huge improvement in our understanding of what parts of the transcriptome are translated. Many alternative open reading frames (ORFs) are now regularly being detected such as out-of-frame, overlapping, upstream or downstream reading frames, and alternative reading frames using non-canonical start codons. Various tools have been developed for the detection of such novel ORFs, but they lack the capacity to visually inspect reads—an important aspect of validation and prediction of translation.

**Results:**

The integrated and visualisation of ribosome profiling and RNA sequencing reads enables discrimination between transcriptional and translational signals, facilitating validation of predicted novel open reading frames. Furthermore, the inclusion of complementary evidence such as proteomic and long-read sequencing enables further validation of predicted novel open reading frames.

**Availability and implementation:**

Here, we present, InspectorORF (https://www.github.com/aylz83/inspectorORF), an R package that readily plots ribosome profiling reads, alongside RNA sequencing reads across transcripts and/or ORFs. Additionally, custom information can be plotted including data from additional conditions and samples, proteomic analyses and reads from long-read sequencing.

## 1 Introduction

The advent of ribosome profiling (Ribo-Seq; Ingolia *et al*. 2011) has enabled the discovery of actively translated open reading frames (ORFs) within expressed transcripts. Since ribosomes advance codon by codon during translation elongation, Ribo-Seq data exhibits a three-nucleotide periodic signal aligned in frame to the ORF. The presence of triplet periodicity is therefore a key indicator of active translation elongation. When mapped to the genome, Ribo-Seq reads enable the identification of actively translated ORFs, typically with canonical start (AUG) and stop (UAG, UGA, or UGA) codons. Alternative methods being translation initiation sequencing (TI-Seq; Ingolia *et al.* 2011) enable the discovery of any translation initiation event of near cognate start codons (CUG, GUG, UUG, AAG or ACG; Andreev *et al.* 2022).

In the human genome, there are currently ∼20 000 annotated protein-coding genes. However, numerous additional types of ORF have been found to be translated (Wright *et al.* 2022). These include (i) upstream ORFs (uORFs), (ii) downstream ORFs (dORFs), (iii) overlapping ORFs, (oORFs), and (iv) out-of-frame ORFs of an existing annotated ORF (Wright *et al.* 2022). Additionally, small ORFs (smORFs) in previously non-coding characterized genes i.e. long non-coding RNAs (lncRNAs), have also been described (Aspden *et al.* 2014, Douka *et al.* 2021). Even in well-characterized model organism genomes, for example, *Saccharomyces cerevisiae* or *Drosophila melanogaster*, many sites of translation remain unannotated and their functions unknown (Rocha *et al.* 2023). Similarly, in prokaryotic genomes like *Escherichia coli*, millions of ORFs have been estimated to exist but only 30%–40% have been characterized as translated (Ochman and Jones 2000), with novel ORFs (unannotated protein-coding regions) regularly found to be translated (Stringer et al. 2022). Therefore, the presence of an ORF, i.e. an in frame start and stop codon, does not necessarily mean the region is translated and producing protein.

Tools to enable the discovery of novel translated-ORFs, from Ribo-Seq data have therefore been developed, such as ORFquant (Calviello *et al.* 2020), RiboTaper (Calviello *et al.* 2016), RiboTish (Zhang *et al.* 2017), or PRICE (Erhard *et al.* 2018). These tools primarily report both annotated and novel ORFs based on Ribo-Seq reads, or a combination of both Ribo-Seq and RNA-Seq. ORFquant and RiboTaper rely on the triplet periodicity that is exhibited within Ribo-Seq reads to determine which frame of a transcript is being translated, if it is genuinely translated by elongating ribosomes. By first building a database of all possible ORFs based on any stop codon which is in-frame to upstream start codons within the transcriptome, the triplet periodicity of Ribo-Seq reads within these ORFs are evaluated by ORFquant/RiboTaper. Translated ORFs are selected by either the first start codon found on the transcript in-frame to the exhibited triplet periodicity, or the first start codon closest to the first set of in-frame reads.

Visual evaluation of Ribo-Seq data for specific transcripts is a crucial and commonly used step in the field, especially when selecting candidates for further wet-lab experimental validation. Despite its importance, this process is often carried out in an *ad hoc* manner across different research groups, limiting reproducibility and consistency. Visual inspection helps assess translation by evaluating whether sufficient triplet periodicity exists within the ORF of interest, distinguishing genuine signal from noise. With noise described as sequencing bias or mapping artifacts which may present as irregular triplet periodicity or extremely sharp peaks at specific codons throughout the ORF. Furthermore, upstream/downstream start codons may be missed by the ORF discovery tool due to the lack of sufficient in-frame reads from incomplete ribosome protected fragment digestion, resulting in missing reads within the data and therefore misdetection of the correct start codon, particularly in the case of upstream canonical start codons. Visualizing the whole transcript can therefore also be beneficial.

Furthermore, tools like ORFquant only use Ribo-Seq data to determine translated ORFs. As not all transcribed genes are translated, and for those that are translated there is considerable variation in signal it is important to evaluate the RNA-Seq reads for a transcript alongside the Ribo-Seq reads for ORFs within these transcripts. Additional omics data, such as (but not limited to) proteomics, long-read sequencing, or modifications counts at specific genomic positions, and metadata, e.g. Kozak scores, can provide critical insight when evaluating whether such ORFs are translated and help increase confidence in identification of the precise ORF being translated.

InspectorORF, an R package, is available at https://www.github.com/aylz83/inspectorORF and has been created to visualize Ribo-Seq data at the transcript level. InspectorORF input is taken directly from RiboTaper and ORFquant output files. InspectorORF can visualize triplet periodicity of an overall transcript (optionally including introns), of a specific ORF of interest, and display alternative upstream/downstream start/stop codons, be it in-frame and out-of-frame or canonical and non-canonical. Being an R package, InspectorORF has the added benefit of (i) bulk generation of plots for exporting, so higher throughput than generating screenshots from IGV, (ii) plotting regions of interest directly integrated into existing pipelines, rather than needing to go back and forth between command line and webtools, and (iii) not needing to upload unpublished data to third party services.

## 2 InspectorORF

**Figure 1:**
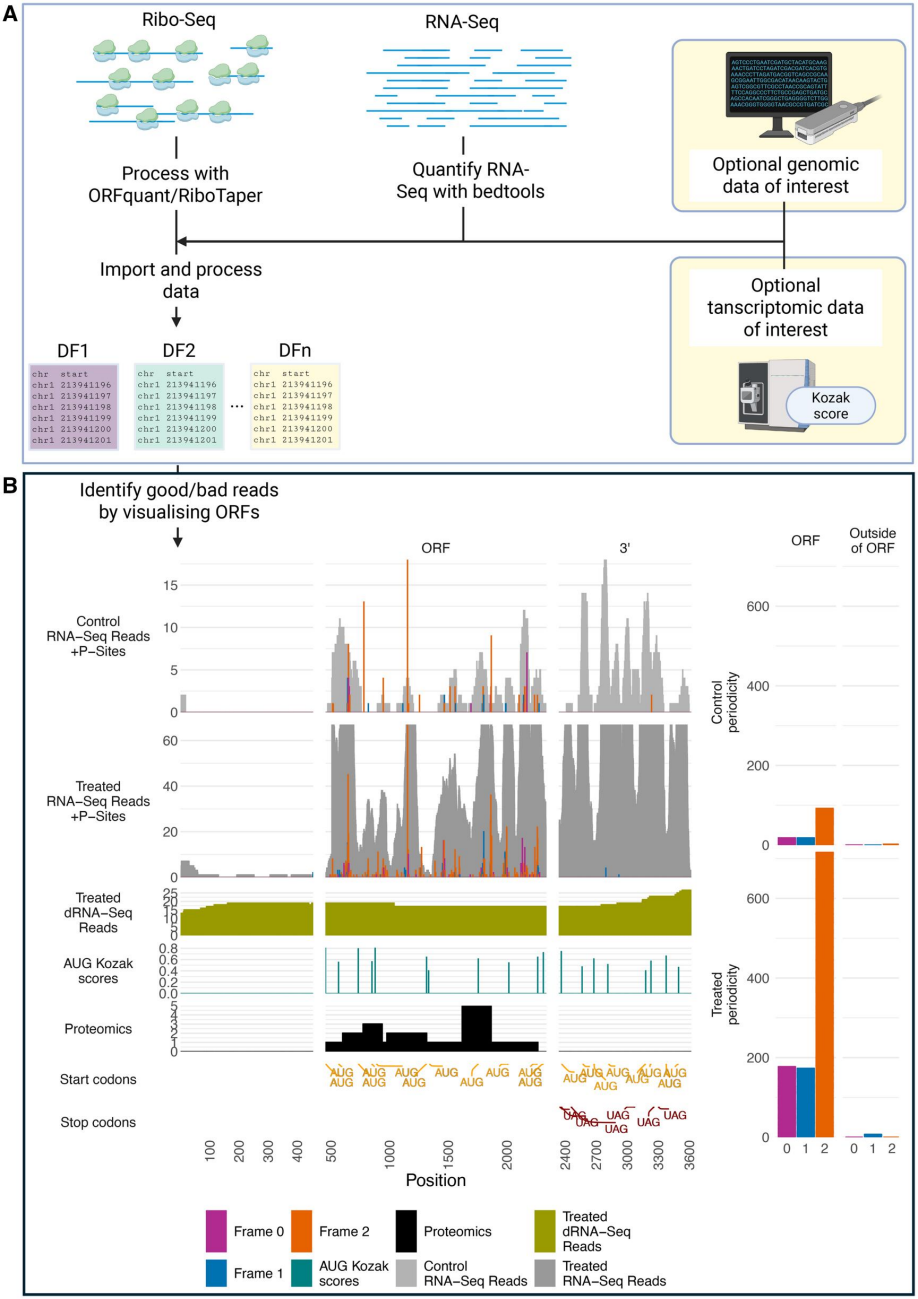
Schematic of information required and process for creating plots with InspectorORF. (A) Input consists of P-sites calculated by ORFquant, RNA-Seq coverage calculated with bedtools and optionally, any additional sequencing data such as additional RNA-Seq data, long-read sequencing data, or coverage calculated from aligned BAM files from high throughput sequencing methods. Importing the genomic data, requesting transcripts of interest resulting in read information being extracted for such transcripts, optionally providing additional transcriptomic level information such as peptide hits from proteomics data mapped to the transcriptome or Kozak scores. (B) InspectorORF plot of ENST00000343702 (NTN4). 5’ UTR, ORF, and 3’ UTR indicated. Limited undifferentiated (control) RNA-Seq and P-site coverage (frame 0, frame 1, frame 2) within the ORF and ORF framing (right) from accession GSE16214 (Douka *et al*. 2021). Limited triplet periodicity observed within the ORF framing plot (right) and no framing observed within the P-site reads for the rest of the transcript 9 (right). Triplet periodicity observed within the differentiated (treated) ORF and ORF framing plot (right, frame 2), and limited reads, in an alternative frame (frame 1) in the rest of the transcript (right). Additional information can be plotted, depending on the type of data present. For example, reads from direct-RNA long-read sequencing, or Kozak scores (scaled 0–1; 0—no Kozak; 1—perfect Kozak sequence) at the ORF start-site, with no upstream Kozaks present. Proteomics evidence of synthesized protein can be plotted, consisting of number of peptide fragment hits for regions of the ORF (proteomics evidence here is synthetic to demonstrate usage). Codons within different regions of the transcript can also be plot, such as all in-frame AUG start codons or stop codons present within the transcript. Created in BioRender.

InspectorORF is an R package with two primary purposes; (i) evaluating Ribo-Seq and RNA-Seq reads for full transcripts and/or (ii) the exploration, identification, and validation of ORFs from Ribo-Seq and RNA-Seq data. Visualization involves two main steps (Fig. 1); (i) The primary pipeline consisting of obtaining genomic data of interest; P-sites calculated by ORFquant or RiboTaper from Ribo-Seq data, RNA-Seq coverage calculated by bedtools, and any additional genomic or transcriptomic information as required, and finally, importing and processing of data with annotation data and (ii) plotting of transcripts or ORFs of interest to evaluate translation, for either single or multiple conditions, optionally requesting framing coverage for the ORF and the remainder of the transcript, and/or in-/out-of-frame start codons of interest. Currently, importing data from RiboTaper is limited to evaluating and visualizing only one condition or replicate at a time.

### 3.1 Importing and processing data

InspectorORF requires reads to be in the form of a BED file or a processed transcript tracks data object, defining each gene or transcript as a track line, followed by two read types at each chromosome position, one for the P-site reads, and a second for RNA-Seq reads (Fig. 1A). Functions are provided for the processing of reads (Fig. 1A) analyzed by RiboTaper and ORFquant, which can either then be read directly by InspectorORF, or saved as a BED file for later use.

Importing data into InspectorORF also requires both a General Transfer Format (GTF) file and a genome sequence file in 2-bit format. The GTF file requires gene id, transcript id, and exon information for extracting reads within exonic regions into transcript variants. The 2-bit file consisting of genome level nucleotide sequences for each gene is required for calculating ORFs within each transcript variant. The 2-bit files can be generated from a FASTA of interest with UCSC’s faToTwoBit application (https://genome.ucsc.edu/goldenpath/help/twoBit.html). Both the GTF and FASTA used for 2-bit generation should be of the same annotation version initially used for ORF discovery with either RiboTaper or ORFquant. Options are provided to retain any reads within the introns of genes.

### 3.2 Additional data features

During the generation of transcript level track information, additional data can be optionally supplied that aligns to transcriptomic level (Fig. 1A), such as Kozak consensus scores (Kozak 1989) for potential start codons along a transcript, translated ORF regions supported by mass spectrometry data, long-read dRNA-Seq data, or any other form of custom metadata consisting of real values at each chromosome position of interest. Helper functions and scripts are provided to aid in the generation of the final transcript’s object/bed file.

### 3.3 Generating transcript/ORF plots

Many ORF discovery tools identify start codons by creating a database of all possible ORFs, followed by indicating candidates as translated based on (i) the start codon nearest to the transcription start site and (ii) the nearest start codon to the first in-frame read. In the case of ORFquant reads require triplet periodicity to be present, and for RiboTish—negative binomial tests. However, the true start codon may be upstream/downstream of this, and in some scenarios, may be a non-canonical start codon. Translation may also initiate at multiple start codons within a transcript (Benitez-Cantos *et al.* 2020). Therefore, InspectorORF has various options to plot a whole transcript with regions of interest (Fig. 1B). Alternatively, for whole transcript plots, panels of exons and/or exons and introns can be generated. One can also specify an ORFs start and stop position in a whole transcript with the coding sequence of interest, resulting in highlighted regions for both 5’ and 3’UTR regions with the ORF of interest, or just the ORF of interest. Annotation of any in-frame or out-of-frame start codons or stop codons can be enabled by specifying codon queries, such as matching the ORFs start/stop codon, as well as alternative (non-)canonical start or stop codons of interest on the same or differing panels, enabling the visualization of reads which may be a result of a different start/stop codon to the one detected. Coupled with the ability to plot additional information such as previously calculated Kozak consensus scores, by providing additional transcript level information during the generation of the transcript tracks, this enables the user to identify possible locations where initiation is more likely to occur, by manual inspection of the data (Fig. 1B).

Where multiple conditions are specified on importing data into InspectorORF, separate graphs of the conditions or samples can be included in the main figure during the generation of the main track information. This results in multiple stacked plots of the same ORF or transcript across multiple conditions, allowing a visual overview of the effect each condition has on translation (Fig. 1B). Comparisons of any triplet periodicity exhibited can also be compared between the P-site coverage within the ORF against the rest of the transcript, which can indicate the ORF is translated if limited P-site reads and triplet periodicity is present outside of the ORF of interest.

Any additional omics data present during the importing of the data into InspectorORF can be visualized. For example, peptide hits from translated ORFs from proteomics evidence can be included in the stacked facet plots, aiding in the determination of regions of the transcript that are translated into protein.

As an ORF supported by Ribo-Seq reads exhibiting sufficient triplet periodicity is one good indicator of an ORF being translated. InspectorORF can therefore plot the reads for each frame as a summarized bar graph for both the overall ORF, as well as reads outside of the ORF (all reads within the transcript minus the reads found within the ORF or region being plot). The triplet periodicity for the rest of the transcript can be a particularly useful metric. A difference between the ORF’s triplet periodicity distribution and the rest of the transcript’s triplet periodicity, would indicate there are ribosomes actively translating the ORF of interest, as opposed to ribosomes or RNA-binding protein complexes simply being associated with the transcript (Fig. 1B).

## Data Availability

InspectorORF is available at https://www.github.com/aylz83/inspectorORF.
